# An Invasive Clonal Plant Benefits from Clonal Integration More than a Co-Occurring Native Plant in Nutrient-Patchy and Competitive Environments

**DOI:** 10.1371/journal.pone.0097246

**Published:** 2014-05-09

**Authors:** Wenhua You, Shufeng Fan, Dan Yu, Dong Xie, Chunhua Liu

**Affiliations:** The National Field Station of Lake Ecosystem of Liangzi Lake, College of Life Science, Wuhan University, Wuhan, P.R. China; Beijing Forestry University, China

## Abstract

Many notorious invasive plants are clonal, however, little is known about the different roles of clonal integration effects between invasive and native plants. Here, we hypothesize that clonal integration affect growth, photosynthetic performance, biomass allocation and thus competitive ability of invasive and native clonal plants, and invasive clonal plants benefit from clonal integration more than co-occurring native plants in heterogeneous habitats. To test these hypotheses, two stoloniferous clonal plants, *Alternanthera philoxeroides* (invasive), *Jussiaea repens* (native) were studied in China. The apical parts of both species were grown either with or without neighboring vegetation and the basal parts without competitors were in nutrient- rich or -poor habitats, with stolon connections were either severed or kept intact. Competition significantly reduced growth and photosynthetic performance of the apical ramets in both species, but not the biomass of neighboring vegetation. Without competition, clonal integration greatly improved the growth and photosynthetic performance of both species, especially when the basal parts were in nutrient-rich habitats. When grown with neighboring vegetation, growth of *J. repens* and photosynthetic performance of both species were significantly enhanced by clonal integration with the basal parts in both nutrient-rich and -poor habitats, while growth and relative neighbor effect (RNE) of *A. philoxeroides* were greatly improved by clonal integration only when the basal parts were in nutrient-rich habitats. Moreover, clonal integration increased *A. philoxeroides*'s biomass allocation to roots without competition, but decreased it with competition, especially when the basal ramets were in nutrient-rich sections. Effects of clonal integration on biomass allocation of *J. repens* was similar to that of *A. philoxeroides* but with less significance. These results supported our hypothesis that invasive clonal plants *A. philoxeroides* benefits from clonal integration more than co-occurring native *J. repens*, suggesting that the invasiveness of *A. philoxeroides* may be closely related to clonal integration in heterogeneous environments.

## Introduction

Clonal integration, through which connected ramets of clonal plants can share water, carbohydrates,nutrients and other substances such as pollutants, diseases, etc. [1]–[3], may improve plants' exploitation of ubiquitous heterogeneous resources, help plants invade new environments and facilitate plants' spatial occupation of new habitats at a local scale [4]. Previous studies have shown that clonal integration may facilitate the colonization and growth of the ramets in heterogeneous habitats with stressful conditions [5], [6], help genets to survive and to recover after severe environmental change [7], [8] and allow for occupation of new space [9]–[11]. These positive effects of clonal integration may increase the performance of clonal plants over non-clonal plants or other clonal plants with little integration [12]. Therefore, increases in performance of clonal plants by clonal integration may affect the growth and reproduction of their co-existence species, and thus influence community structure and ecosystem function [13], [14].

Plant invasions pose a great threat to biodiversity and global ecosystem stability [15], [16]. Many of the most notorious alien invasive plants have the capacity for vigorous clonal propagation [17], [18]. Some studies have suggested that the invasiveness of alien clonal plants may be closely correlated to clonal integration [4], [19], [20]. However, to our best of knowledge, few studies have focused on how clonal integration affects invasion of alien invasive clonal plants to native plant communities, but see [9], [21]–[24]. Therefore, a better understanding of different clonal integration effects between alien invasive and native clonal plants when competing with each other is both scientific and practical interests.

Previous studies about the effects of clonal integration on performance of clonal plants when competing with neighbors were with inconsistent results [9]. Clonal integration had no significant effects on competitive ability of several terrestrial or amphibious plants [9], [14], [21], [25], but it did increase growth of several salt marsh plants for below-ground resources, the competitive ability of *Solidago canadensis* against interspecific neighbors and the invasion of smooth brome clones to northern fescue prairies [26]–[28]. However, none of these studies had investigated how clonal integration affected the growth of the neighboring vegetation (competitors), but see [9]. In the study of Pennings and Callaway (2000), the results showed that physiological integration played different roles in six salt marsh clonal plants with recipient ramets in different microhabitats or competing with co-existing neighbors in different status (clipped or kept intact). Moreover, several studies have addressed the effects of clonal integration on intra- or interspecific competition of recipient ramets [9], [29], [30]. Unfortunately, all these studies ignored the status of other connected ramets (donor ramets) and the environments which in, as clonal plants often experience small-scale spatial heterogeneity duo to large clonal systems [31]. For instance, clonal integration may increase the competitive ability of ramets when other connected ones were in resource-rich patches, because more subsidy could be supplied by donor ramets in non-limiting resource environments, which may facilitate the invasion of the clonal plants to neighboring communities. But to our knowledge, no studies have tested this. Furthermore, in a field experiment, Peltzer (2002) found that clonal integration did not alter the effects of competition from neighboring vegetation for *Populus tremuloides*, however, competition greatly improved the survivorship of *Populus* ramets after 2 years. Therefore, the importance of clonal integration in competition urgently needed for further research [21].

In heterogeneous habitats consisting of a mixture of rich and poor resource patches, via clonal integration, clonal plants can alter biomass allocation and divert more biomass to shoots or roots for acquisition of more abundant resource, and exploration of more favourable space, a phenomenon called ‘division of labour' [2], [32], [33]. This pattern of biomass allocation is different from that used by non-clonal plants, or clonal plants grown in homogeneous conditions [2], [32]. In particular, the relationship between plant photosynthetic efficiency and clonal integration has not been widely studied [9], [31]. Photosynthetic efficiency can be estimated by measuring chlorophyll fluorescence [34]. A sensitive indicator of plant photosynthetic performance derived from the parameters of chlorophyll fluorescence is the maximum quantum yield of photosystem II (*F*_v_/*F*_m_), which usually significantly decreases when plants are faced with environmental stress [31], [35]. Environmental stress on ramets may be alleviated by clonal integration, which may markedly lower the negative effects of stress on *F*_v_/*F*_m_ [9], [11]. Moreover, photosynthetic activity, measured in terms of the effective quantum yield of PSII (Yield), is closely related to plant performance. Biomass allocation and photosynthetic efficiency can both contribute to the performance of clonal plants when exposed to competitive stress, however, our understanding of their responses to clonal integration for invasive plants remains limited [9], [11].

Therefore, to test different effects of clonal integration on one exotic invasive and one native clonal plants, we conducted a greenhouse experiment to investigate responses of growth, biomass allocation, photosynthetic performance and relative neighbor effects (RNE, used to indicate the plants' competitive ability) of two stoloniferous clonal plants, *Alternanthera philoxeroides* (invasive), *Jussiaea repens* (native) to clonal integration when competing with competitors (neighboring vegetation) in China. We used a factorial design with resource availability, stolon severing and competition with neighboring vegetation as factors. Specifically, we hypothesize 1) that clonal integration will improve growth and competitive ability of the two clonal plants, especially when donor ramets are in nutrient-rich habitats, 2) that clonal integration will modify biomass allocation of the two plants grown with competitors. Based on the theory of labour division [32], we predict that, through clonal integration, plants will allocate more biomass to leaves if the belowground competition is more severe and will allocate more biomass to roots if aboveground competition is more severe, 3) that clonal integration will enhance the photosynthetic performance and buffer the decrease in *F*_v_/*F*_m_ of the two plants in competitive environments, especially when donor ramets are in nutrient-rich habitats, 4) that *A. philoxeroides* will benefit from clonal integration more than *J. repens*, in terms of competitive ability, photosynthetic performance and capacity of labor division, and 5) that clonal integration of these two clonal species will suppress the growth of neighboring vegetation due to competition with apical ramets.

## Materials and Methods

### Plant material

*A. philoxeroides* is a serious economic and environmental clonal weed which originates from Parana River region of South America and now invades may countries in the world [36], [37]. In China, *A. philoxeroides* has invaded varied ecosystems and caused great economic and environmental problems, and it is listed as one of the 16 worst alien invasive weeds [38]. *J. repens* is a rooted emergent stoloniferous clonal plants and a fast-proliferating species in wetlands, naturally distributed in central and south China [39]. In natural environments, these two species often co-exist in diverse habitats that from wet to aquatic in south China.

In early May 2010, source material of *A. philoxeroides* and *J. repens* was collected from at least five locations at least 15 m apart in each of two wetlands in Liangzi Lake in the Hubei province of China (N 30°05′–30°18′, E 114°21′–114°39′). Given that genetic diversity of wetland clonal plants is relatively low [40], especially for *A. philoxeroides* in China [41], different populations of each species were assumed to belong to the same genet. Then plants from different locations were mixed and propagated in the greenhouse. After two weeks of adaptive culture, about 600 tip cuttings of each plant were collected and planted vertically into 12 plots (30 cm diameter ×15 cm height) with soil (TN 2.94 mg/g, TP 0.13 mg/g) from the lake side of Liangzi Lake. Ten days later, a homogeneous subset of 480 vigorously growing plants of each species were selected for this experiment.

### Ethics Statement

Plant material used in this experiment was collected from natural plant populations at the National Field Station of Freshwater Ecosystem of Liangzi Lake (N 30°05′–30°18′, E 114°21′–114°39′). Both of the plant species were common and naturally distributed in this area. No specific permissions were required for these locations. This study did not involve endangered or protected species.

### Experimental design

**Figure 1:**
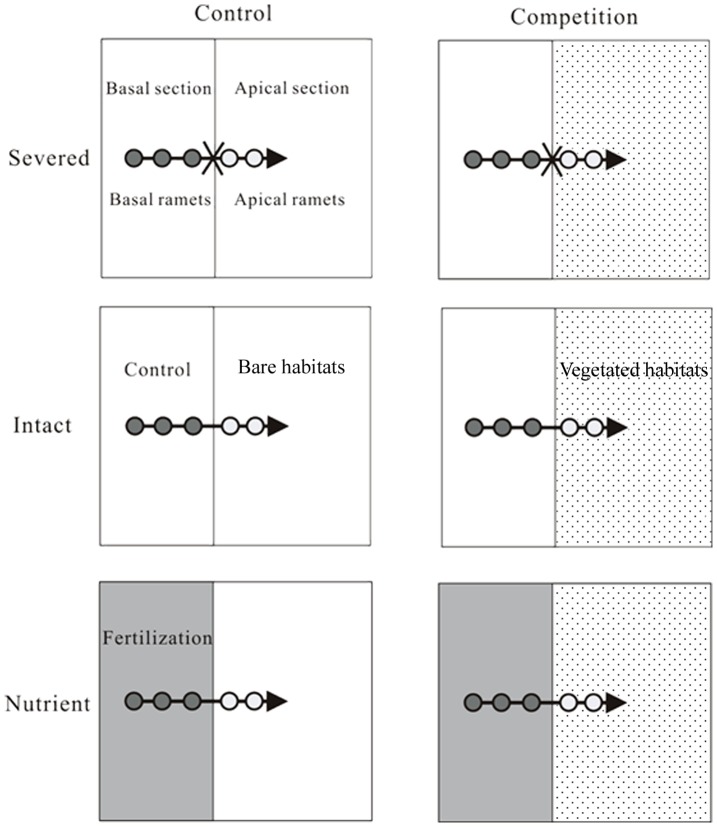
Schematic representation of the experimental design. Clonal fragments of the invasive plant *A. philoxeroides* or native plant *J. repens*, each consisting of three basal ramets (dark grey circles) and two apical ramets (light gray circles) with a stolon apex (horizontal arrow), were grown either with (competition) or without (control) competitive vegetation (*J. repens* or *A. philoxeroides*, spot-shadow) and with stolon connections between basal and apical ramets were either intact or severed (fork). Three integration treatments were used as follows: severed (stolon connections severed by the scissors), intact (stolon connections kept intact) and nutrient (stolon connections kept intact and with basal ramets in fertilized habitats).

The growth experiment was conducted in a glasshouse under natural sunlight (about 14/10 day/night cycle) and ambient temperature at the National Field Station of the Lake Ecosystem of Liangzi Lake, Wuhan University. The experiment was conducted with a factorial design involving competition (without or with vegetation for control or competition treatment) and integration treatments (stolon connections were severed, intact or intact and with basal ramets in nutrient-rich patches, for severed, intact or nutrient treatment) (Fig. 1). The tested plants used in this experiment were 24 similar-sized clonal fragments (tip cuttings, 14.33±0.15 cm in length, 0.62±0.034 g in dry mass for *A. philoxeroides*; 15.02±0.21 cm in length, 0.78±0.041 g in dry mass for *L.repens*; means ± SE), each consisting of a stolon with five ramets for each species. No differences between treatments were detected in initial size of this plants (*P*>0.05 for both species, One-way ANOVA). Each clonal fragment was divided into two parts, one termed as ‘basal part’ consisting of three relatively old ramets (close to the mother ramets) and the other as ‘apical part’ consisting two relatively young ramets (distal to the mother ramets) and a stolon apex.

There were 28 plastic containers (50×50×25 cm; length×width×height), each having two separated sections in this experiment for each species (see Fig. 1). The basal section was 20 cm long and the apical section was 30 cm long. Resources (nutrients and water) and roots in the two sections did not interfere with each other. All the containers in both sections were filled with a mixture of sand and lake mud at a volume ratio of 3∶1. To create highly fertilized soil patches, 12 containers were filled with the same mixture and 5 g slow-release fertilizer (Osmocote®, N–P–K: 16–8–12, 6 month) in the basal section. On June 10th 2011, the apical sections of 16 containers were planted vertically with cultured plant fragments of each species (monoculture) in the glasshouse to mimic natural plant populations (vegetated habitats), with a density of 200 plants m^−2^ (30 plants in each apical section) for each species. The remaining 12 containers were kept with apical sections bare.

On July 5th 2011, 24 clonal fragments of each species were horizontally positioned in 24 containers (12 with and 12 without competitive vegetation in apical sections), the remaining 4 containers with competitive vegetation were used as a control for plant population growth without competition. For each clonal fragment, three ramets of basal part were placed within basal section of a container and the other two ramets and apex of the apical part were within the apical section of the same container. The stolon of the apical ramets was anchored to the soil surface to facilitating rooting. Six days later, when the clonal fragments were successfully rooted, the stolon connections between the apical and basal parts were severed in 8 containers, while the other 16 ones were kept intact (see Fig. 1). The experiment was ended on September 10th 2011. The experimental units were randomly repositioned every two weeks to avoid the effects of possible environmental heterogeneity (such as light), and watered every other day to maintain the soil in the containers at wet condition. The mean light intensity in the greenhouse was 800–1200 µmol m^−2^ s^−1^, and the mean air temperature was 20–28°C during the experimental period.

### Measurements

One week before harvesting the plants, the minimum (*F*_0_) and the maximum (*F*_m_) fluorescence yield were measured for a fully developed, healthy leaf on the second-youngest of the ramets in each apical plant after a dark adaptation (shaded by leaf folders) of at least 20 minutes sufficient for photosystem II (PSII) reaction centers to open by a portable chlorophyll fluorometer (DIVING-PAM, Walz, Effeltrich, Germany) with the saturation pulse method [34]. The maximum quantum yield of PSII (*F*_v_/*F*_m_) was calculated as (*F*_m_ – *F*_0_)/*F*_m_. The effective quantum yield of PS II (Yield) was calculated as (*F*_m_′–*F*_t_)/*F*_m_′, where *F*_m_′ is defined as the maximal fluorescence yield reached in a pulse of saturating light after a actinic light pulse of 120 µmol m^−2^ s^−1^ for 10 seconds, and *F*_t_ is the fluorescence yield of the leaf at that photosynthetic photon flux density [31], [35].

At harvest, the number of ramets and leaves were counted, and the total stolon length, total leaf area (Li-3100 Area Meter, Li-Cor, USA) were measured for the apical parts of all treatments. The ramets in the apical part of the two clonal species were then harvested and separated into leaves, stolons and roots, and their biomass was determined after drying at 70 °C for 72 h. Neighboring vegetation (entire plants including roots) in the apical sections of the container for each species were also harvested and their dry mass was also determined in the same way.

The relative neighbor effect (RNE) was calculated to measure the competitive intensity [42]. The RNE of plant was calculated as (*C* - *A*)/max (*C*, *A*), where is *A* the mean biomass of plant across replicates without competition, *C* is biomass of plant with competition, and max (*C*, *A*) is the larger value between *A* and *C*. Usually. The values of RNE range from -1 to 0, and the greater the values are, the smaller the neighbor's effects is [9]. So, a significantly larger RNE with than without stolon connection treatments indicates clonal integration facilitates plant's competitive ability.

### Data analysis

All data were log transformed to meet assumptions of normality and homoscedasticity before analysis. One-way ANOVA was used to test whether total biomass of vegetation (competitors) in the apical section for each species differed among the four treatments (no competition; competition with severed stolon connection; competition with intact stolon connection; competition with intact stolon connection and basal parts in nutrient-rich sections). Two-way ANOVA was used to assess the effects of integration treatments (severed, intact and nutrient) and competition on photosynthetic performance (*F*_v_/*F*_m_ and Yield) of the two species in the apical section. Two-way multivariate analysis of variance (MANOVA) was employed to investigate the global effects of integration treatments and competition on growth measures (total biomass, ramet number, stolon length, leaf number and leaf area) and biomass allocation pattern (biomass allocation to leaves, stolons and roots) of both species in the apical parts, and corresponding univariate analyses were also conducted. If a significant treatment effect was detected, post-hoc pair-wise comparisons of means were made to examine differences between treatments using Studentized Tukey's HSD for multiple comparisons. The differences of RNE values among the integration treatments were tested by one-way ANOVA followed by Duncan tests. Statistical significance was assigned at a *P*<0.05. All data analyses were performed using SPSS 18.0 (SPSS, Chicago, IL, USA).

## Results

### Growth and the relative neighbor effect (RNE)

**Figure 2:**
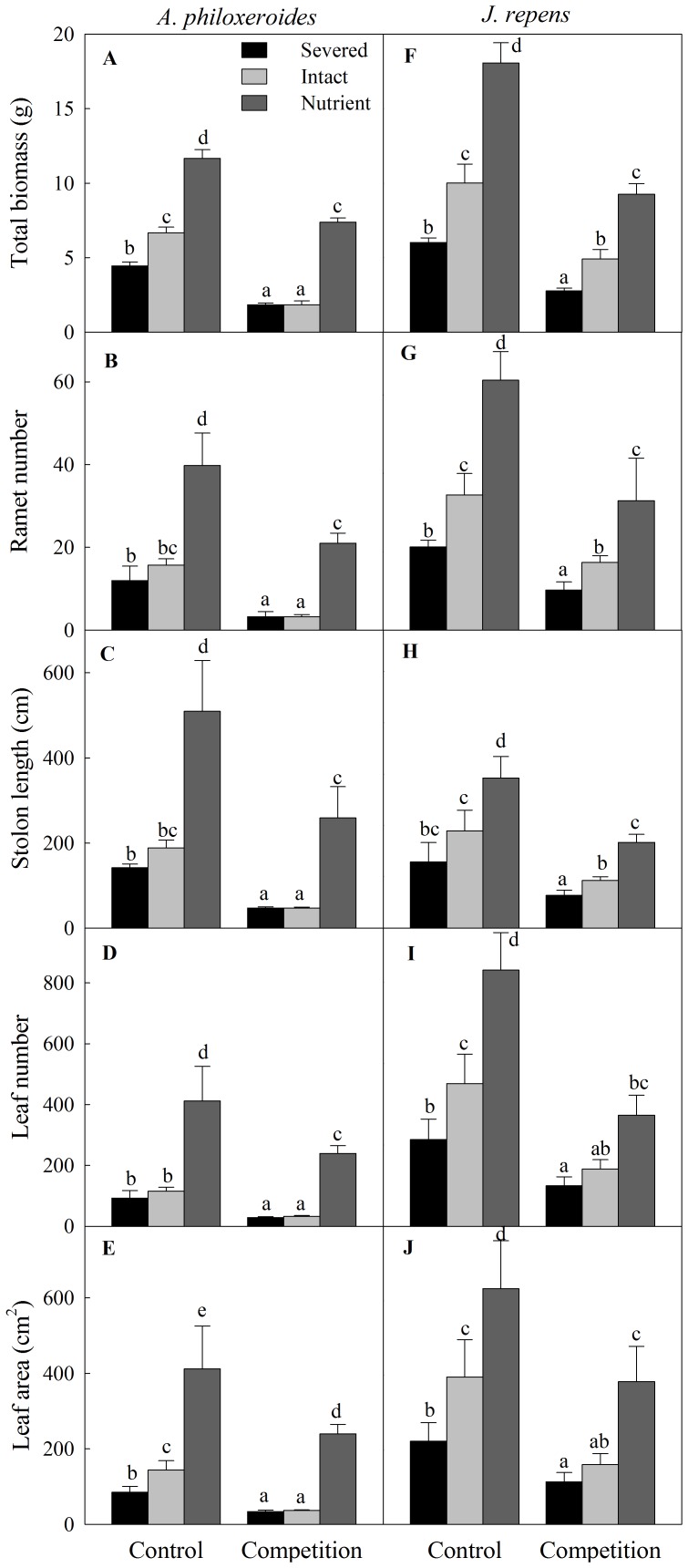
Effects of integration treatments and competition on growth measures of the two clonal plants. Total biomass, ramet number, stolon length, leaf number and total leaf area of the invasive plant *A. philoxeroides* (left: A, B, C, D, E) or native plant *J. repens* (right: F, G, H, I, J) in the apical sections, grown either with or without competitive vegetation (*J. repens* or *A. philoxeroides*) in three integration treatments. Data indicate the means ± SE. Bars sharing the same letter are not significantly different at *P* = 0.05.

Integration treatments and competition had significant effects on growth of both clonal species in the apical sections, and their interaction was also significant for *A. philoxeroides* but not for *J. repens* (Table 1). Competition greatly reduced the growth measures (including total biomass, number of ramets and leaves, stolon length and total leaf area) of the two clonal species (Table 1, Fig 2). Without competition, clonal integration greatly improved the growth of both of these two species in the apical sections, especially when the basal parts of the clonal fragments were in nutrient-rich patches (Fig 2A, B, C, D, E). However, with competition, clonal integration had no significant effect on the growth of *A. philoxeroides* but greatly enhanced that when its basal parts were in nutrient-rich sections (Fig 2A, B, C, D, E). For *J. repens*, the responses of the growth to integration treatments with competition were similar to that without competition (Fig 2F, G, H, I, J).

**Table 1:** Summary of MANOVA and univariate ANOVA for effects of integration treatments and competition on growth measures of the two clonal plants in the apical sections.

Multivariate test statistics								
	*A. philoxeroides*				*J. repens*			
Source	Wilk's Lambda	*F*	d.f.	*P*	Wilk's Lambda	*F*	d.f.	*P*
Integration (I)	0.008	29.37	10,28	**<0.001**	0.018	17.93	10,28	**<0.001**
Competition (C)	0.015	180.23	5,14	**<0.001**	0.037	73.37	5,14	**<0.001**
I×C	0.079	7.14	10,28	**<0.001**	0.403	1.61	10,28	0.16

Univariate test statistics										
	*A. philoxeroides*					*J. repens*				
Source	Total	Stolon	Ramet	Leaf	Total	Total	Stolon	Ramet	Leaf	Total
	biomass	length	number	number	leaf area	biomass	length	number	number	leaf area
Integration (I)	611.68***	101.10***	202.22***	250.56***	275.19***	288.89***	71.32***	59.50***	60.74***	47.53***
Competition(C)	88.55***	146.37***	260.16***	181.63***	208.87***	404.84***	95.78***	134.55***	113.31***	52.91***
I×C	67.03***	8.79**	9.54**	10.15**	18.45***	3.22	0.40	2.45	0.32	1.35
d.f.	5,18	5,18	5,18	5,18	5,18	5,18	5,18	5,18	5,18	5,18

Significant *P*-values are presented in bold.

Values give *F*; symbols give *P*: * P<0.05; ** P<0.01; *** P<0.001.

**Figure 3:**
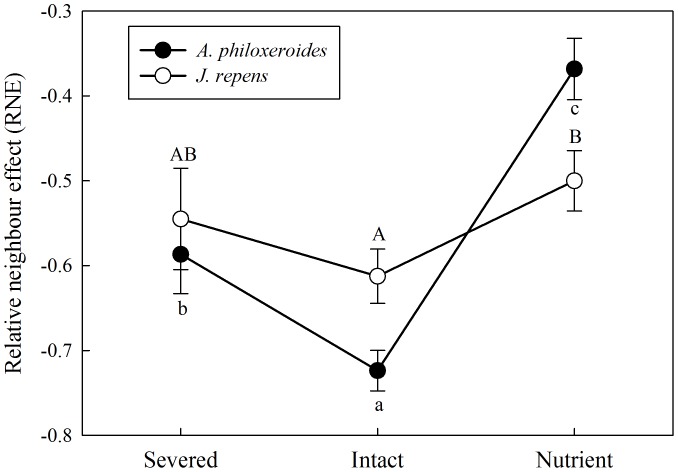
Effects of integration treatments on the relative neighbour effect (RNE) of the two clonal plants. The relative neighbour effect (RNE) of the invasive plant *A. philoxeroides* and native plant *J. repens* in the apical sections in three integration treatments. Data indicate the means ± SE. Bars sharing the same letter are not significantly different at *P* = 0.05.

Integration treatments (severed, intact and nutrient) significantly affected the relative neighbor effect (RNE) of the two clonal species in the apical parts (*F*_2,11_ = 96.14, *P*<0.001 for *A. philoxeroides*; *F*_2,11_ = 6.24, *P* = 0.02 for *J. repens*). Clonal integration significantly decreased the RNE of *A. philoxeroides* but greatly increased that in nutrient treatment (Fig. 3). The RNE of *J. repens* had a decreasing trend with the stolon connection intact and an increasing trend in nutrient treatment but not significantly (Fig. 3).

### Biomass allocation pattern

**Figure 4:**
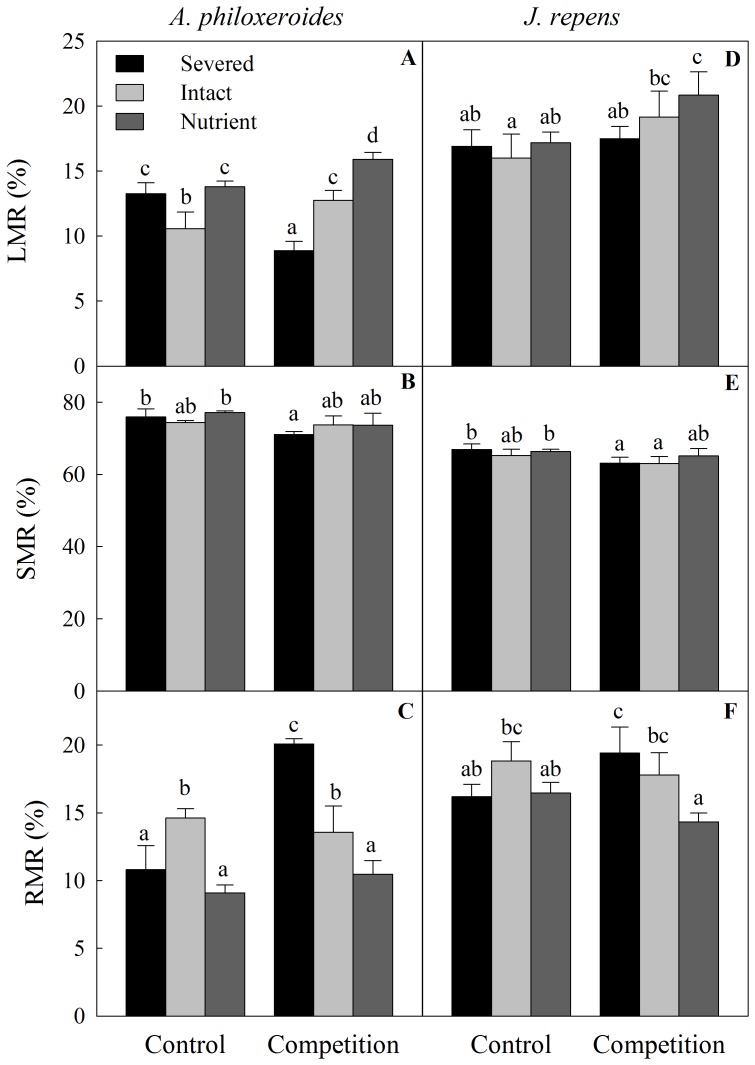
Effects of integration treatments and competition on biomass allocation of the two clonal plants. Biomass allocation (LMR, leaf mass ratio; SMR, stolon mass ratio; RMR, root mass ratio) of the invasive plant *A. philoxeroides* (left: A, B, C) or native plant *J. repens* (right: E, F, G) in the apical sections, grown either with or without competitive vegetation (*J. repens* or *A. philoxeroides*) in three integration treatments. Data indicate the means ± SE.

Integration treatments, competition and their interaction significantly affected the biomass allocation of both species in the apical sections (Table 2). Clonal integration significantly increased biomass allocation of *A. philoxeroides* to the roots and decreased that to the leaves without competition, whereas it decreased biomass allocation to the roots and increased that to the leaves with competition (Table 2, Fig. 4A, C). However, when the basal parts of *A. philoxeroides* were in nutrient-rich sections, clonal integration increased biomass allocation to the leaves and decreased that to the roots whether when the apical parts of plants were with competition or not (Table 2, Fig. 4A, C). Biomass allocation to the stolons of both species were not affected by clonal integration but was significantly larger when the apical ramets were grown with rather than without competition (Table 2, Fig. 4B, E). Effects of integration treatments and competition on apical parts of *J. repens* were similar to that of *A. philoxeroides*, although the trend was less obvious (Table 2, Fig. 4D, F).

**Table 2:** Summary of MANOVA and univariate ANOVA for effects of integration treatments and competition on biomass allocation of the two clonal plants in the apical sections.

Multivariate test statistics								
	*A. philoxeroides*				*J. repens*			
Source	Wilk's Lambda	*F*	d.f.	*P*	Wilk's Lambda	*F*	d.f.	*P*
Integration (I)	0.074	14.30	6,32	**<0.001**	0.414	2.95	6,32	**0.021**
Competition (C)	0.302	12.35	3,16	**<0.001**	0.472	5.96	3,16	**0.006**
I×C	0.080	13.50	6,32	**<0.001**	0.457	1.61	6,32	**0.039**

Univariate test statistics						
	*A. philoxeroides*			*J. repens*		
Source	LMR	SMR	RMR	LMR	SMR	RMR
Integration (I)	50.54***	1.98	47.00***	3.17	1.87	11.23**
Competition(C)	0.10	14.17**	41.07***	15.89**	12.57**	0.05
I×C	43.33***	2.45	39.13***	2.38	1.21	9.26**
d.f.	5,18	5,18	5,18	5,18	5,18	5,18

Significant *P*-values are presented in bold.

LMR: leaf mass ratio, SMR: stolon mass ratio, RMR: root mass ratio.

Values give *F*; symbols give *P*: * P<0.05; ** P<0.01; *** P<0.001.

### Photosynthetic performance

**Figure 5:**
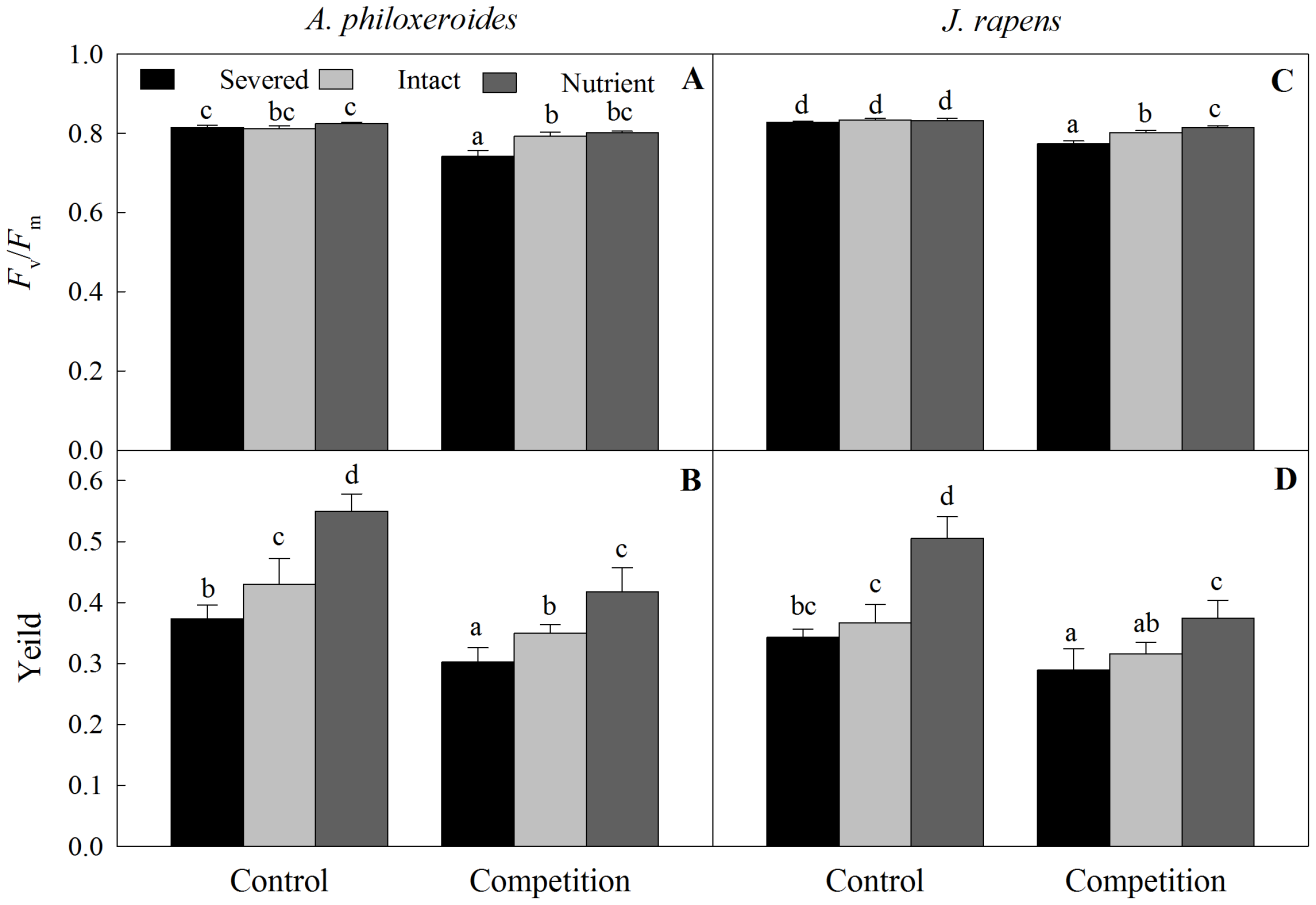
Effects of integration treatments and competition on photosynthetic performance of the two clonal plants. The maximum quantum yield of photosystem II (*F*_v_/*F*_m_) and the effective quantum yield of PSII (Yield) of the invasive plant *A. philoxeroides* (left: A, B) or native plant *J. repens* (right: C, D) in the apical sections, grown either with or without competitive vegetation (*J. repens* or *A. philoxeroides*) in three integration treatments. Data indicate the means ± SE.

Integration treatments and competition significantly affected the photosynthetic performance (*F*_v_/*F*_m_ and Yield) of both of the two clonal plants, and their interaction was also significant in *F*_v_/*F*_m_ but not in Yield (Table 3). Competition greatly reduced the value of *F*_v_/*F*_m_ of the two species in the apical sections, especially when the stolon connections were severed (Fig. 5A, C). Clonal integration markedly increased the value of *F*_v_/*F*_m_ of the two species, especially when their basal parts were in nutrient-rich sections (Fig. 5A, C). The Yields of the two plants were both significantly reduced by competition (Fig. 5B, D), but greatly enhanced by clonal integration, especially when their basal parts were in nutrient-rich sections (Fig. 5B, D).

**Table 3:** Two-way ANOVA for effects of integration treatments and competition on the maximum quantum yield of photosystem II (Fv/Fm) and the effective quantum yield of PSII (Yield) of the two clonal plants in the apical sections.

	*A. philoxeroides*		*J. repens*	
Source	*F*_v_/*F*_m_	Yield	*F*_v_/*F*_m_	Yield
Integration (I)	*F*_2,42_ = 62.74***	*F*_2,42_ = 55.34***	*F*_2,42_ = 85.56***	*F*_2,42_ = 75.09***
Competition (C)	*F*_1,42_ = 142.21***	*F*_1,42_ = 78.63***	*F*_1,42_ = 262.33***	*F*_1,42_ = 64.72***
I×C	*F*_2,42_ = 50.24***	*F*_2,42_ = 2.21	*F*_2,42_ = 50.70***	*F*_2,42_ = 3.04

Values give *F*; symbols give *P*: * P<0.05; ** P<0.01; *** P<0.001.

### Growth of neighboring vegetation

Total neighboring vegetation biomass of the two species both had no significant differences among all the treatments (*F*_3,15_ = 0.87, *P* = 0.39 for *A. philoxeroides*; *F*_3,15_ = 0.48, *P* = 0.70 for *J. repens*). Total biomass in the apical sections for each species in four treatments (no competition; competition with severed stolon connection; competition with intact stolon connection; competition with intact stolon connection and basal parts in rich-patches) were 74.72±2.13 g, 76.33±3.21 g, 74.54±1.94 g and 73.52±3.17 g for vegetation of *A. philoxeroides*; and 89.38±3.67 g, 91.87±0.70 g, 93.59±2.97 g and 89.81±2.99 g (means ± SE) for that of *J. repens* respectively.

## Discussion

Clonal integration improved the growth and photosynthetic performance, modified biomass allocation of both the introduced, invasive species *A. philoxeroides* and the co-occurring native species *J. repens* in nutrient-patchy and competitive environments. These results suggest that clonal integration is very important for both species when faced with competition in heterogeneous habitats [9]. However, some differences were observed in these two stoloniferous clonal plants.

### Effects of clonal integration on growth and competitive ability

Without competition, clonal integration significantly improved the growth of both clonal species in the apical sections, especially when their basal parts were in nutrient-rich sections. This result occurred most likely because the well-established ramets in the basal sections supported the growth of the interconnected young apical sections and facilitated the production of new tissue due to acropetal (from basal ramets to apical ramets) translocation of carbohydrates via clonal integration [9]. The results agree with those obtained in previous studies on several other clonal plants including terrestrial plants [43], [44], amphibious plants [9] and submerged aquatic plants [10], which showed that clonal integration facilitates establishment of newly produced ramets, improves growth of adult ramets and helps genets to occupy open space. This phenomenon was more pronounced when basal ramets were in nutrient-rich patches, probably because more subsidy was provided by the basal parts via clonal integration due to source-sink relationship [31], [45], [46]. These observations indicate that clonal integration is critical in allowing these two clonal species to explore new open space and rapid expansion, especially in heterogeneous habitats, which is a well-known mechanism to allow stoloniferous and rhizomatous plants to forage for resources over large areas [45], [46].

When competing with neighboring vegetation, growth measures of both plants were markedly suppressed by competition, suggesting that strong interspecific competition in this experiment occurred in the apical parts of both plants. Interestingly, stolon connection had no effect on growth of *A. philoxeroides* with competition, while it greatly increased growth of that in the nutrient treatment. Moreover, clonal integration decreased the RNE of *A. philoxeroides* in intact treatment and greatly increased that in nutrient treatment, suggesting that clonal integration improved the competitive ability of *A. philoxeroides* only when the basal parts were in nutrient-rich conditions. The reason might be that ramets of *A. philoxeroides* were sophisticated (selective) and relatively independent. Therefore, few ramets were placed in the habitat with severe competition (less resources available in apical sections) and more ramets were placed in relatively more favourable conditions (more resources available in bare basal sections) [47], [48]. Actually, more branches and biomass were observed in the basal sections (data not shown). However, when the basal parts of the plant were in nutrient-rich sections, strong intra-competition existed because of dense plant population due to vigorous growth in nutrient-rich habitats at the end of experiment. Nutrient-rich habitats may have equal or even less suitability compared to poor habitats because overcrowding reduces suitability [31]. To avoid self-shading, clonal integration enhanced the competitive ability and facilitated the invasion of the apical ramets of *A. philoxeroides* into neighboring vegetation [49]. For *J. repens*, with competition of *A. philoxeroides* vegetation, clonal integration promoted the growth of apical parts of plant in both stolon connection treatments (intact and nutrient treatments). In addition, clonal integration had no significant effects on the RNE of the apical ramets whether in intact treatment or in nutrient treatment. These results suggest that native *J. repens* may be more dependent on physiological integration and may share resources to a higher degree [50]. Thus, when faced with severed competition, growth and spread of the invasive *A. philoxeroides* would in general benefit more from clonal integration than native co-occurring *J. repens*, because: 1) in relatively poor habitats, clonal integration may preferentially allow the ramets of *A. philoxeroides* to escape from competitive stress and explore other open space to rapid expansion [9]; 2) in resource-rich patchy habitats, clonal integration may enhance the its competitive ability and facilitate the invasion of *A. philoxeroides* to neighboring vegetation [22].

### Effects of clonal integration on biomass allocation pattern

Biomass allocations of both plants were significantly influenced by clonal integration which is consistent with previous findings for many other clonal plants [9], [32], [51]. Without competition, clonal integration increased biomass allocation of *A. philoxeroides* to roots at the expense of that to leaves, however, when its basal parts were in nutrient-rich sections, clonal integration reversed this trend of biomass allocation. The results occurred most likely because soil resources were relatively more limiting for expansion of the ramets in the apical sections without competition [9]. For the connected apical ramets, the required carbohydrates could be transported efficiently from the basal ramets, so that relatively more biomass could be allocated to roots in order to improve the growth of the whole ramet system in the apical parts [9]. Additionally, when the donor ramets were in nutrient-rich habitats, due to source-sink relationship [31], [52], nutrient is not limiting resources and ramets in apical parts can allocate more fraction of biomass to aboveground (leaves and stolons) to rapid spread and occupation of new habitat.

With competition, however, clonal integration greatly increased biomass allocation to leaves and decreased that to roots, especially when basal parts of plants were in nutrient-rich sections. This might be because under severe competition, allocating more biomass to leaves can help apical ramets to harvest relatively more abundant light (by placing above the canopy of the dense vegetation), whereas poor soil resources (due to dense roots of competitive vegetation) could be compensated by basal ramets through clonal integration [9], [53], especially when basal ramets were placed in resourceful conditions. These observations suggest that biomass allocation of invasive plant *A. philoxeroides* in present study agrees with the theory of labor division theory in clonal plants [32]. That is, young ramets in the apical sections explore locally most abundant resource (light) and receive mineral nutrients and water from older ramets in the basal sections via xylem, while carbohydrates can be imported or produced locally and even exported [46]. In this situation, environmentally induced labor division occurred in the apical ramets, as two essential resources (light and nutrients and/or water) negatively correlated due to competition by neighboring vegetation [54]. Biomass allocation pattern of *J. repens* was similar to that of *A. philoxeroides* but with less significance, suggesting that different integration strategies may occur in these two clonal plants when faced with competition. For instance, differences in extent of integration or degree of physiological integration in these two species may exist in heterogeneous environments [45], [55]. Indeed, invasive clonal plants with stoloniferous perennial growth are considered to be conferred with the ability of rapidly covering areas via changing biomass allocation through clonal integration [9], [11]. However, this needs further in-depth investigation.

### Effects of clonal integration on photosynthetic performance

In favourable conditions, the value of *F*_v_/*F*_m_ for most plant species ranges from 0.8 to 0.84 [9], [11]. Without competition, *F*_v_/*F*_m_ values of ramets of both plants in all clonal treatments were within the normal range of healthy plants and exhibited no significant differences among all the treatments, while growing with competitive vegetation greatly decreased *F*_v_/*F*_m_ of both plants to the degree outside the normal range, suggesting that severe competition imposed stress on them. However, the decrease of plants' *F*_v_/*F*_m_ values was markedly alleviated by stolon connections, especially when the donor ramets were in nutrient-rich sections, allowing the ramets to maintain *F*_v_/*F*_m_ values within the normal range. Therefore, the results suggest that clonal integration significantly buffered plants against competitive stress and significantly increased plant photosynthetic performance. Previous studies [9], [31] also found that clonal integration significantly alleviated the decrease in *F*_v_/*F*_m_ of ramets grown in soils with heavy metals or with severe competition by neighboring plants. Moreover, photosynthetic capacity, measured in terms of the effective quantum yield of PS II (Yield), was significantly improved by clonal integration and nutrient addition in both plants. The responses of Yield values were closely correlated with the growth of apical ramets in response to clonal integration under competition, suggesting that clonal integration improved growth of both plants in patchy habitats when competing with competitors mainly by increasing photosynthetic efficiencies [31]. The benefit of clonal integration in terms of physiological traits (photochemical activity determined by *F*_v_/*F*_m_ and Yield) supported our hypothesis and reinforced the capacity of division labor in these two plants, as an increase in *F*_v_/*F*_m_ (and Yield) and a reduction of biomass allocation to the roots were observed in integration treatments, which could be interpreted as a specialization for aboveground resources. These results suggest that division of labor in stoloniferous clonal plants can happen both at morphological and physiological level [54]. However, no differences were observed in these two plants, indicating that differences in growth and division of biomass between the two species may due to different resource-sharing strategies mediated by clonal integration [45], [55].

### Effects of clonal integration on neighboring vegetation

Interestingly, total biomass of neighboring vegetation of both species was not affected by the presence of apical ramets, suggesting that competition treatments in present study did not suppress growth of plant populations. This is most likely because competition between apical ramets and competitive vegetation was asymmetrical because of low density of apical ramets in this experiment and their biomass was too small to influence the plant community [10]. This observation was supported by the fact that biomass of apical ramets in both plants with competition was sharply decreased to less than 30% as compared with that without competition. Therefore, even though clonal integration greatly improved the growth of plants in the apical sections under competition, their relatively small biomass contributed little to affect the plant community due to relatively short experimental duration (12 weeks). It can be expected that roles of clonal integration may be more important with longer experimental duration.

## Conclusions

Overall, when competing with neighboring vegetation, clonal integration greatly improved growth and photosynthetic performance of both species when the connected basal ramets were in nutrient-rich habitats, suggesting that clonal integration is important for both species in nutrient-patchy and competitive environments. However, ramets of the invasive *A. philoxeroides* were more sophisticated and independent than the co-occurring native *J. repens* when faced with competition. Moreover, under competitive environments, changes in biomass allocation of *A. philoxeroides* through clonal integration was more significant than that of *J. repens*, although biomass allocation of both species well conformed to the theory of labor division, suggesting that different integration strategies may occur in these two clonal plants. These observations supported our hypothesis that invasive *A. philoxeroides* may benefit from clonal integration more than co-occurring native *J. repens*, indicating that invasiveness of *A. philoxeroides* may be closely related to clonal integration in heterogeneous environments. However, future comparative research is needed on additional species pairs in order to assemble conclusive evidence on the importance of integration for invasive and native species.
